# Research hotspots and frontiers in depression and anxiety in multiple sclerosis

**DOI:** 10.1097/MD.0000000000047476

**Published:** 2026-02-06

**Authors:** Rongrong Yuan, Linlin Zhang, Qin Yang

**Affiliations:** a Rehabilitation Medicine Department, Nantong Fourth People's Hospital, Nantong, China.

**Keywords:** anxiety, bibliometrics, data visualization, depression, multiple sclerosis

## Abstract

**Background:**

Multiple sclerosis (MS) is a chronic central nervous system disease. Depression and anxiety are common psychiatric symptoms in MS patients. This study conducted a bibliometric analysis of the literature in the field of MS depression and anxiety to reveal current research hotspots and guide future research directions.

**Methods:**

This study used VOSviewer 1.6.18 and Citespace 6.4.R1 to analyze co-occurrence and co-citation of publications, countries/institutions, authors, keywords, and references in the Web of Science Core Collection related to MS depression and anxiety. The research trajectory was described.

**Results:**

This study included 4156 publications. The number of publications has gradually increased since the database was established. Among the 86 countries conducting research in this field, the United States published the most articles, and the University of Washington being the most prolific institution. This makes the United States the core research country in this field. Ruth Ann Marrie from the University of Manitoba published the most articles. Among the 10,270 identified keywords, citation bursts were observed in terms such as “exercise,” “mental health,” “life,” and “adults.” Among the top 10 highly cited articles, 2 were epidemiological studies, with the most cited article being one on the prevalence of depression and anxiety in MS published by Ruth Ann Marrie.

**Conclusion:**

Over the past 20 years, research on depression and anxiety related to MS has increased significantly. The bibliometric analysis results indicate that the epidemiology, etiology, cognitive impairment, and exercise therapy of MS depression and anxiety may be the hotspots for future research.

## 1. Introduction

Multiple sclerosis (MS) is a chronic inflammatory demyelinating disease of the central nervous system (CNS), characterized by inflammation and demyelination within the CNS.^[1,2]^ The etiology is not fully understood but is likely related to genetic, environmental, and viral infection factors. It predominantly affects young and middle-aged individuals, with a higher prevalence in females.^[3]^ Common symptoms include limb weakness, sensory abnormalities, visual disturbances, muscle stiffness, and fatigue.^[4]^

Psychiatric symptoms in MS are also of significant concern. Depression and anxiety in MS can manifest early in the disease course, even preceding typical symptoms.^[5]^ The exact etiology remains unclear, but it is hypothesized that the interaction between physical functional changes in MS patients and social, psychological, and inflammatory factors may contribute to the development of these symptoms.^[6]^ Depression is one of the main determinants of quality of life (QoL) in MS patients, potentially leading to suicidal ideation and affecting adherence to disease-modifying treatments.^[7]^

While research on depression and anxiety in MS has seen a notable increase in recent years, there remains a deficiency in comprehensive reviews and analyses of the studies within this domain. Traditional literature reviews are adept at summarizing and synthesizing existing research findings; however, they often fall short in identifying emerging research trends, analyzing international collaborative networks, and highlighting underrepresented areas. In contrast, bibliometrics, through the quantitative analysis of bibliographic data, offers insights into the dissemination of scientific knowledge, the structure of research activities, and the impact of academic achievements.^[8]^ This approach provides researchers with a broader and more systematic perspective. For instance, bibliometric analysis can elucidate the evolution of research hotspots, uncover collaborative networks across countries and institutions, and identify areas that have not yet garnered adequate attention. Such information is invaluable for guiding future research directions.

## 2. Materials and methods

### 2.1. Data preparation

**Figure 1.**
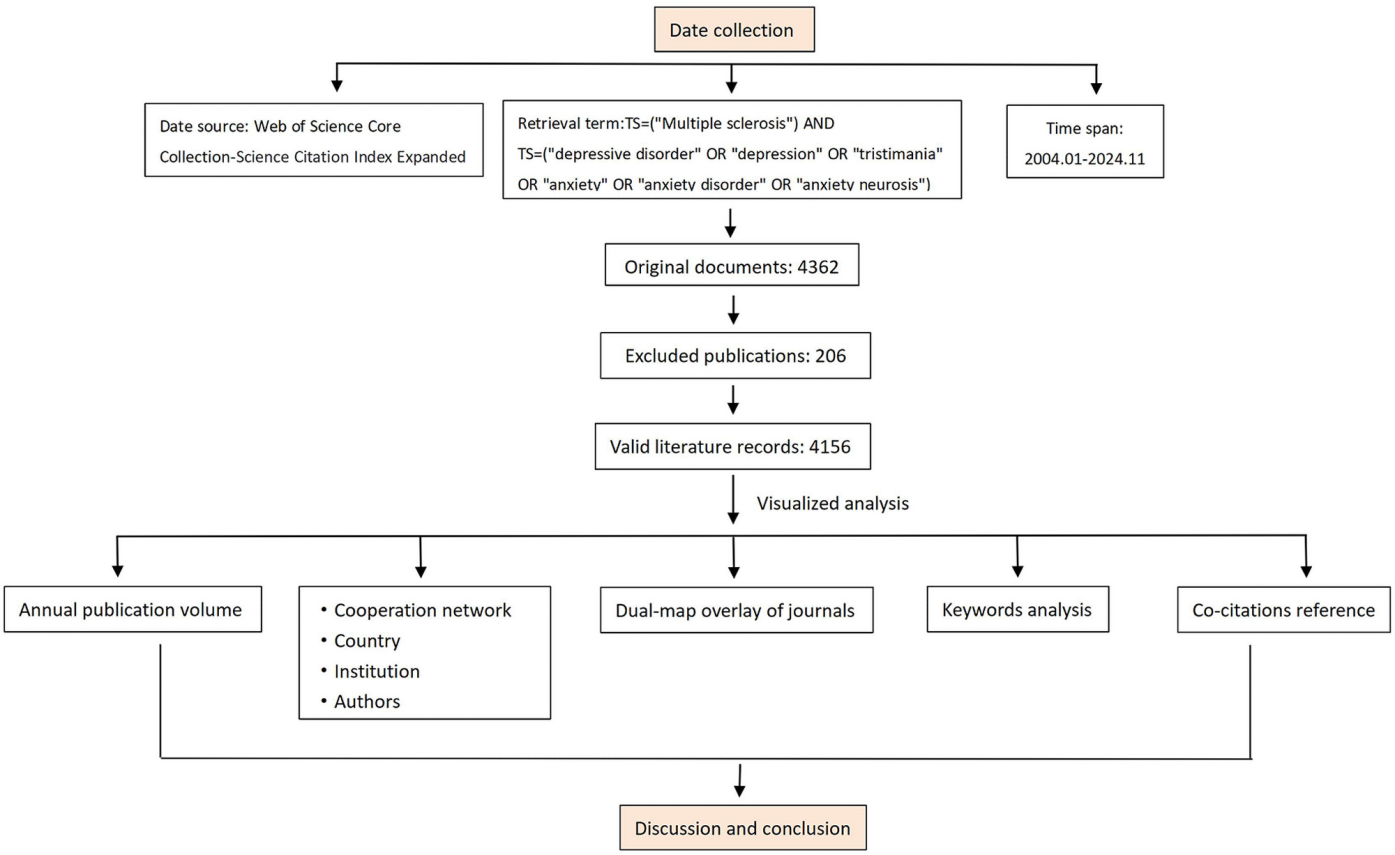
Flowchart for screening the included literature.

Web of Science Core Collection is a high-quality literature database within WoS, providing up-to-date and reliable information. To ensure both literature quality and the completeness of citation information, we exclusively retrieved recent publications related to MS from the Science Citation Index Expanded, a core database within Web of Science. The search strategy was: TS=(“Multiple sclerosis”) AND TS=(“depressive disorder” OR “depression” OR “tristimania” OR “anxiety” OR “anxiety disorder” OR “anxiety neurosis”). The inclusion criteria were: time span: January 2004–November 2024; no language restrictions; and article type limited to “Article.” A total of 4362 publications were initially retrieved. During the data screening process, articles unrelated to MS depression and anxiety were manually excluded. Two researchers independently completed the retrieval and screening of the literature. Ultimately, 4156 publications were available for analysis. In the data analysis process, keywords, countries, or institutions with the same meaning were merged using Citespace 6.4.R1 and VOSviewer 1.6.18. The literature screening process is shown in Figure 1.

### 2.2. Analysis tool parameter settings

In this study, we employed 2 bibliometric analysis tools – VOSviewer (version 1.6.18) and CiteSpace (version 6.4.R1) – to systematically process and visualize data retrieved from the Web of Science Core Collection. In CiteSpace, the following parameters were configured: time slice = 1 year; node types = Country, Institution, Author, Keyword, and Reference; clustering algorithm = Log-Likelihood Ratio ; threshold = top 50 items/slice; network pruning = Pathfinder and “pruning the merged network” to optimize network structure and highlight core nodes. In VOSviewer, the minimum occurrence threshold for keyword co-occurrence analysis was set at 10, and the minimum citation count for co-citation analysis was set at 5. Additionally, during the data-preprocessing stage, both tools were used to merge and standardize keywords, author names, and institutional affiliations, eliminating duplicates and variants and thus enhancing the accuracy and consistency of the analyses.

### 2.3. Tool analysis result notes

Node size corresponds to occurrence frequency: the larger the node, the greater the activity and influence of that element (country, institution, author, or keyword) within the field. The links between nodes denote collaboration, co-occurrence, or co-citation relationships; link thickness and color indicate connection strength and cluster affiliation. Identical colors indicate that the elements have been grouped into the same cluster by the log-likelihood ratio algorithm, signifying similarity in research themes or collaborative teams.

## 3. Results

### 3.1. Publication trends

**Figure 2.**
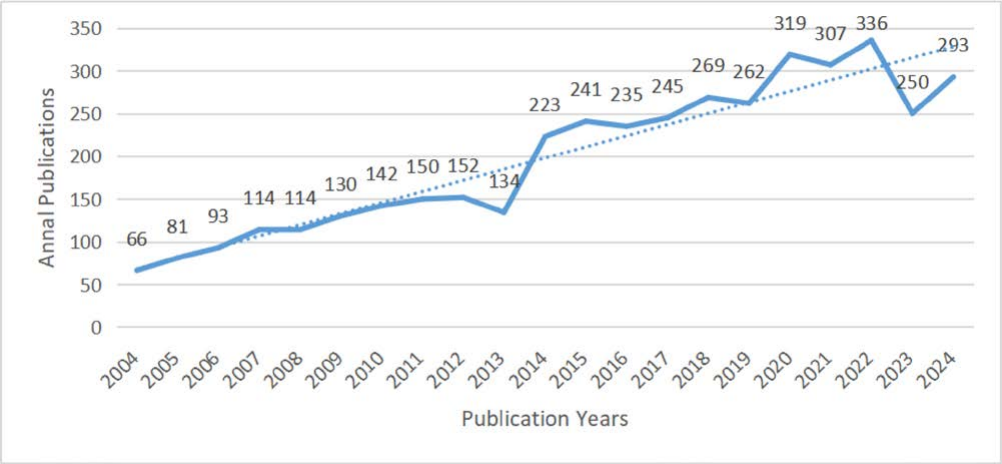
The annual number of global publications and citations.

The number of publications on MS depression and anxiety has gradually increased in recent years (Fig. 2). The trend can be divided into 2 phases: the first phase from 2004 to 2013, during which the number of publications grew steadily; the second phase from 2014 to the present, during which the number of publications increased rapidly with significant fluctuations, peaking in 2022 at 336 publications.

### 3.2. Country and institution analysis

**Figure 3.**
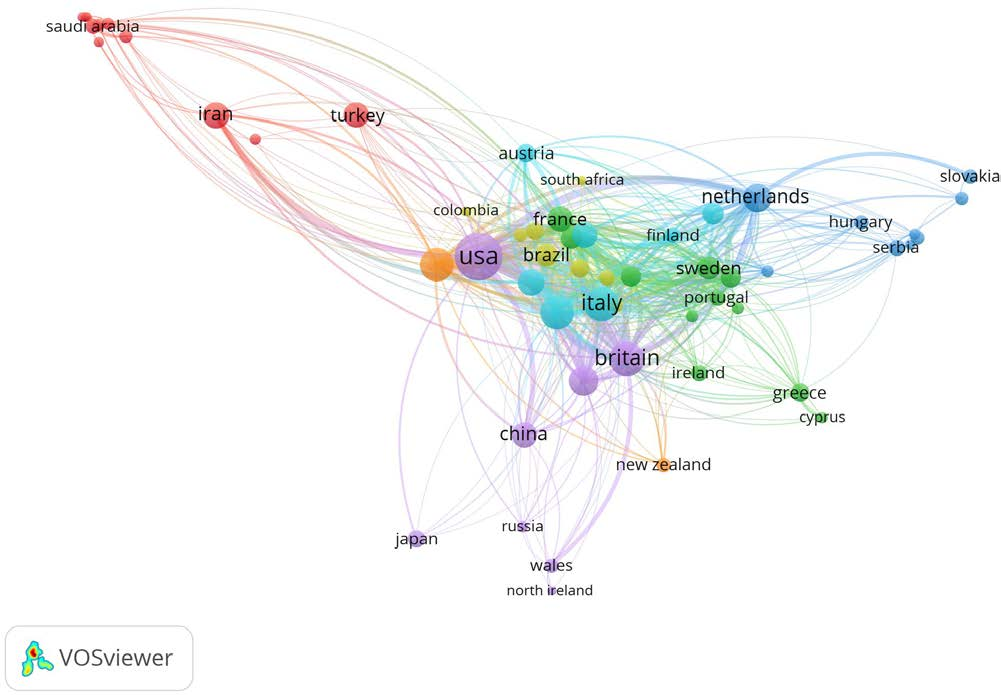
Collaborating countries. The size of the colored nodes indicates the number of published articles. The connections between nodes represent the strength of relationships between countries/regions. The color of the connecting lines indicates clustering.

A total of 86 countries have conducted research on MS depression and anxiety in recent years (Fig. 3, Table 1). The United States ranked first with 1205 publications, accounting for nearly one-third of the total publications, making it the core research country in this field. This dominant position suggests a strong research infrastructure and substantial funding in the United States, which likely drives global research trends in this field. Following the United States, the countries with the highest number of publications were Italy (425), Britain (405), Germany (389), and Canada (363). The research in this field is highly concentrated in these countries, which together account for 50% of the total publications. Average citations per paper reflect research quality and impact. Notably, Switzerland – ranked eighth in output – achieves the highest average (63). Other European countries, such as the United Kingdom and Italy, also record high averages. In contrast, Asian countries like Iran and China lag behind in this metric, likely owing to weaker research infrastructures and a later start in the field. In terms of international collaboration, the United States is the most widely collaborating country, with close cooperation with the top 10 publishing countries. This extensive collaboration network likely facilitates the rapid dissemination of new findings and fosters innovation in the field.

**Table 1:** The top 10 institutes and countries in terms of publication count and centrality.

Rank	Country	Count	Citations	Total link strength	Average citation	Institution	Count	Citations	Total link strength	Average citation
1	USA	1205	47,310	693	39	University Washington	101	2681	41	27
2	Italy	425	18,104	349	43	University Melbourne	92	2385	77	26
3	Britain	405	16,624	498	41	University Manitoba	83	3158	178	38
4	Germany	389	19,019	424	49	University Toronto	83	3009	110	36
5	Canada	363	14,315	291	39	University California	81	3875	69	48
6	Australia	236	6991	220	30	University Calgary	79	3069	134	39
7	Netherlands	231	12,596	270	55	Karolinska Institute	77	3247	51	42
8	Switzerland	175	11,053	322	63	University Illinois	62	1929	58	31
9	Iran	167	3197	73	19	University Alabama Birmingham	59	1507	99	26
10	China	159	2769	68	17	Kings College London	57	1920	28	34

**Figure 4.**
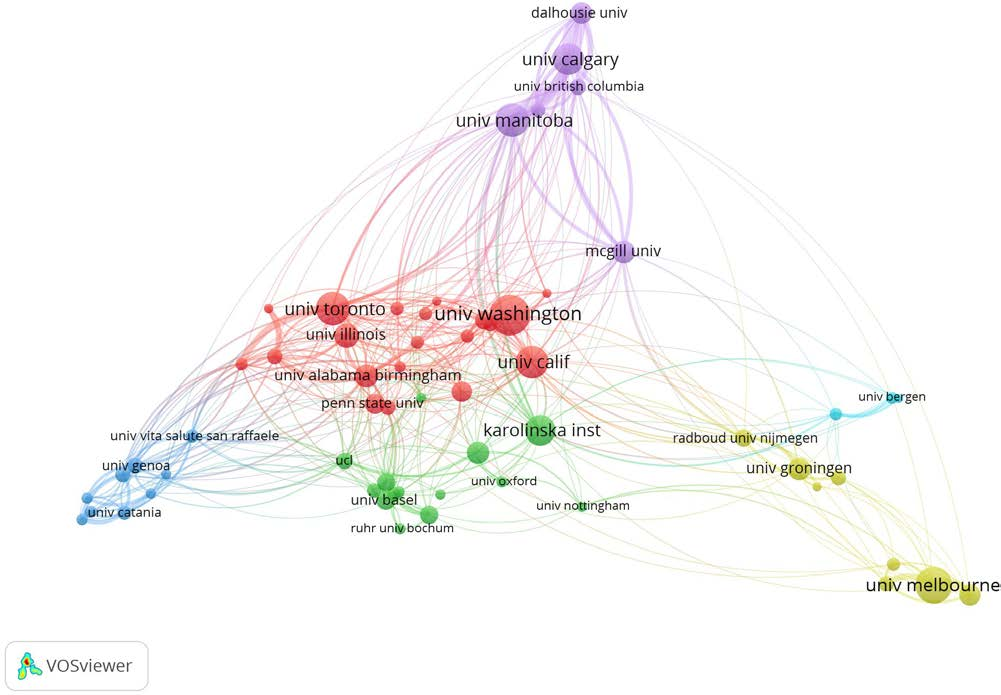
Collaborating institutions. Figure highlights the extensive collaboration network among leading institutions. The University of Washington, the University of California, and the University of Toronto are central nodes, indicating their active involvement in collaborative research. The clustering of institutions around these hubs suggests a dynamic and interconnected research environment, facilitating the rapid dissemination of new findings.

Institutional Co-occurrence Analysis reveals that 4873 institutions have contributed to research in this field. The University of Washington leads with 101 publications, highlighting its significant role in driving research efforts. Other top contributors include the University of Melbourne (92), University of Manitoba (83), University of Toronto (83), and University of California (81). Notably, the University of Manitoba not only records the highest total link strength but also ranks among the top in average citations per paper (38), underscoring its position as one of the most influential institutions in the field. The co-occurrence analysis using VOSviewer (Fig. 4) illustrates the collaborative networks among these institutions. The analysis identifies 6 distinct clusters of collaboration, centered around high-output institutions such as the University of Washington, the University of Melbourne, and the University of Manitoba. These clusters suggest a dynamic and interconnected research environment, where leading institutions play a pivotal role in fostering collaboration and advancing the field. This collaborative structure likely accelerates the dissemination of new findings and enhances the overall impact of research on MS depression and anxiety.

### 3.3. Author co-occurrence analysis

**Figure 5.**
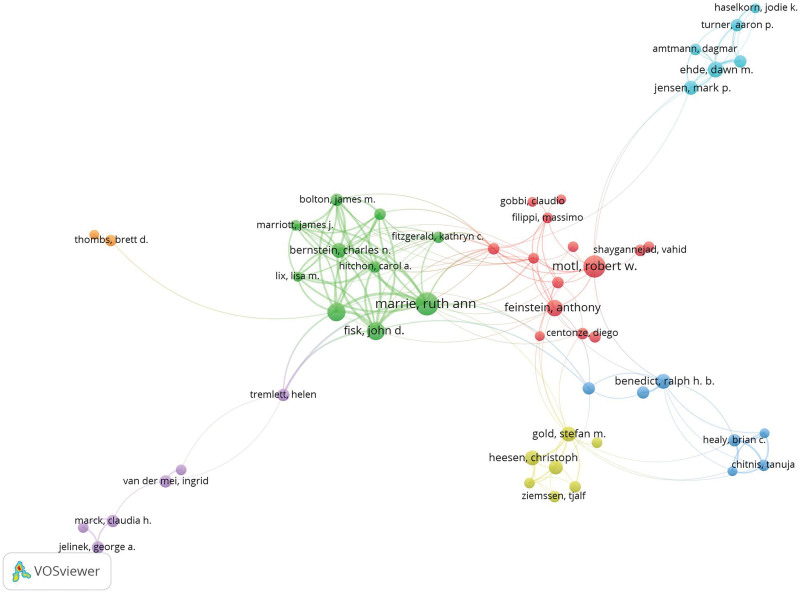
Author co-occurrence. Figure presents the co-occurrence network of authors, with Ruth Ann Marrie from the University of Manitoba as a central figure. This network demonstrates strong internal connections within collaborative teams and highlights the significant contributions of key authors. The clustering of authors around Marrie and other prominent researchers indicates a collaborative and dynamic research landscape.

The co-occurrence network map can display the relationships between different authors (Fig. 5, Table 2). A total of 18,935 authors have contributed to MS mental health research. Among all authors, the most prolific is Marrie, Ruth Ann from the University of Manitoba, who also leads in total citations. Her extensive work on the diagnosis and epidemiology of depression and anxiety in MS underscores her pivotal role in shaping the field. Next, when examining *H*-index and average citations per paper (ACP), Bernstein, Charles N – ranked 10th in publication count – holds the highest *H*-index (108), yet his ACP ranks relatively low. This discrepancy suggests that Bernstein’s primary research focus may lie outside the psychiatric aspects of MS.

**Table 2:** The top 10 authors and their organizations.

Rank	Author	Institute	Count	Citations	*H*-Index	Average citation
1	Marrie, Ruth Ann	University of Manitoba	71	2625	77	36
2	Motl, Robert	University Alabama Birmingham	65	1540	81	23
3	Patten, Scott	University of Manitoba	44	1785	102	40
4	Fisk, John	Dalhousie University	42	1551	65	36
5	Feinstein, Anthony	University Toronto	36	1354	55	37
6	Ehde, Dawn	University Washington	34	912	53	27
7	Arnett, Peter	Penn State University	31	445	40	14
8	Heesen, Christoph	University Medical Centre Hamburg Eppendorf	31	742	50	24
9	Benedict, Ralph	Buffalo General Hospital	28	1563	78	56
10	Bernstein, Charles	University Manitoba	28	655	108	23

In contrast, Patten, Scott B not only boasts an *H*-index exceeding 100 (102) but also records the highest ACP (40), indicating that his studies are of exceptionally high quality and have attracted the greatest attention within the field.

In Figure 5, the color of the nodes represents author clusters, with nodes of the same color forming a collaborative network. The network map also identifies 7 distinct collaborative networks centered around key figures such as Marrie, Ruth Ann; Motl, Robert W; Thombs, Brett D; Tremlett, Helen; Gold, Stefan M; Benedict, Ralph H.B.; and Ehde, Dawn M. These networks demonstrate strong internal connections and interconnections between teams, suggesting a collaborative and dynamic research environment. This collaborative structure likely accelerates the exchange of ideas and the development of new research directions.

### 3.4. Keyword analysis

#### 3.4.1. Keyword co-occurrence analysis

**Figure 6.**
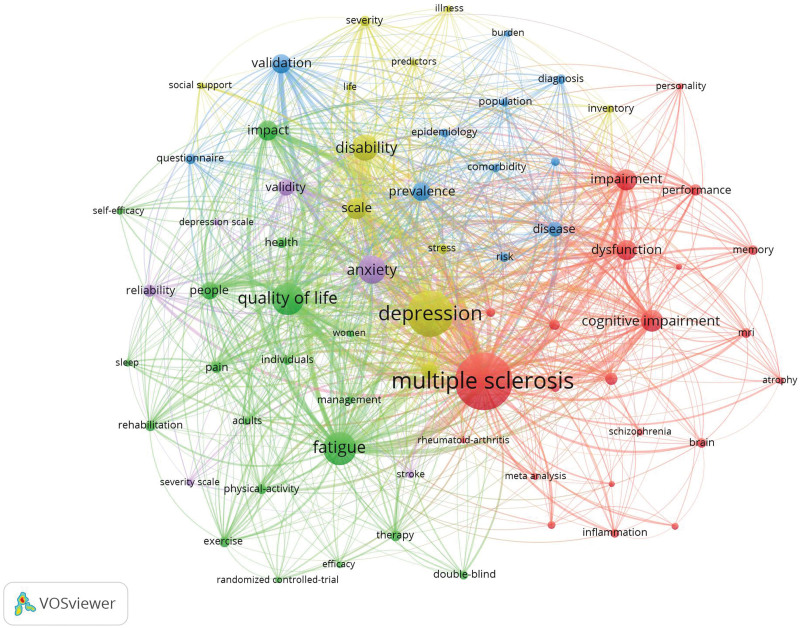
Co-occurring keywords. Figure reveals the co-occurrence of keywords, highlighting the main research themes in the field. The high frequency of terms such as “fatigue,” “quality of life,” and “cognitive impairment” indicates a strong focus on patient-centered outcomes.

Keyword co-occurrence analysis reflects the research focus of the field by identifying terms that frequently appear together in publications. This method helps to uncover the main themes and connections within the literature (Fig. 6). A total of 10,270 keywords appeared, and using citation frequency as the main observation, similar keywords were merged, and subject terms were excluded. The top 10 most frequent keywords were: fatigue, QoL, disability, cognitive impairment, impairment, scale, impact, dysfunction, validation, prevalence. The prominence of these keywords suggests that current research is heavily focused on understanding the impact of MS on patients’ daily lives and cognitive functions. This focus reflects a growing recognition of the importance of holistic patient care, extending beyond traditional medical treatments to include mental health and QoL.

#### 3.4.2. Keyword clustering

**Figure 7.**
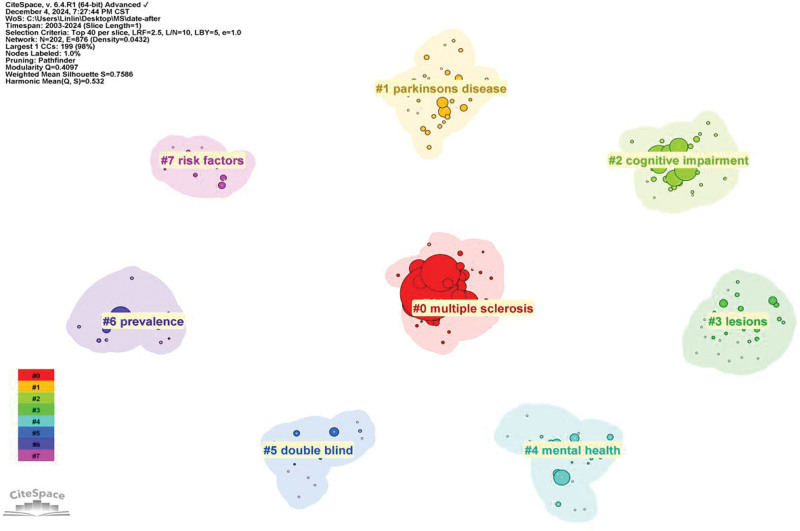
Clustering map of keywords.

Keyword clustering is a technique used to group similar keywords into clusters based on their co-occurrence patterns. This helps to identify distinct research topics and their development trends. Using Citespace, we created a clustering map (Fig. 7) to visualize these clusters (Fig. 7). The *Q* value (0.4097) and *S* value (0.7586) measure the reliability of the clustering results. A *Q* value >0.3 and an *S* value >0.7 indicate that the clustering results are credible. The analysis successfully identified 8 distinct clustering labels: #0 multiple sclerosis, #1 parkinsons disease, #2 cognitive impairment, #3 lesions, #4 mental health, #5 double blind, #6 prevalence, #7 risk factors. These clusters highlight the evolving focus of research from basic disease mechanisms to broader mental health implications, indicating a shift towards more comprehensive and patient-centered research approaches.

#### 3.4.3. Keyword citation burst analysis

**Figure 8.**
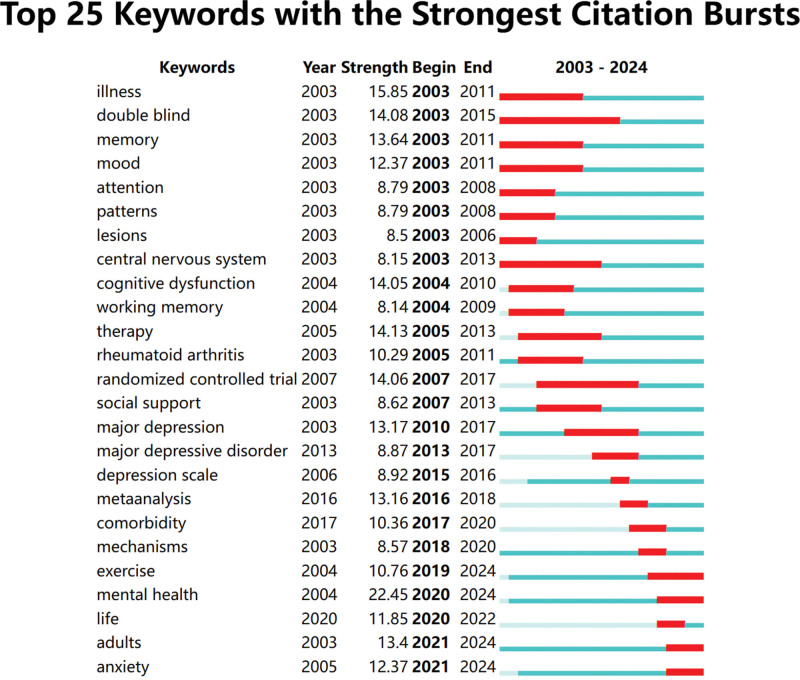
The top keywords with the strongest citation bursts. The keyword citation burst analysis method was employed. In keyword burst analysis, “start” and “end” denote the time period of the burst. “Strength” refers to the intensity of the burst, representing the credibility over time.

Citation burst analysis identifies keywords that have experienced a sudden increase in citations over a specific period. This indicates emerging research hotspots and trends. Using Citespace, a keyword citation burst map was drawn (Fig. 8). The research themes in this field have evolved from the recognition of disease symptoms and exploration of mechanisms, to the development of pharmacological treatments and diagnostic criteria, and finally to the social impacts related to the disease. The citation burst analysis can be divided into 3 phases: the first phase from 2003 to 2011, during which the burst keywords mainly focused on “illness, double blind, memory, mood, attention, patterns, lesions, CNS, cognitive dysfunction, working memory, therapy, rheumatoid arthritis, randomized controlled trial, social support”; the second phase from 2012 to 2017, during which the burst keywords mainly focused on “major depression, major depressive disorder, depression scale, meta-analysis, comorbidity, mechanisms”; the third phase from 2018 to the present, during which the burst keywords mainly focused on “exercise, mental health, life, adults, anxiety.” In this phase, research on MS depression and anxiety has become more in-depth, extending to the impact on patients’ QoL.

### 3.5. Document analysis

#### 3.5.1. Co-citation analysis

**Figure 9.**
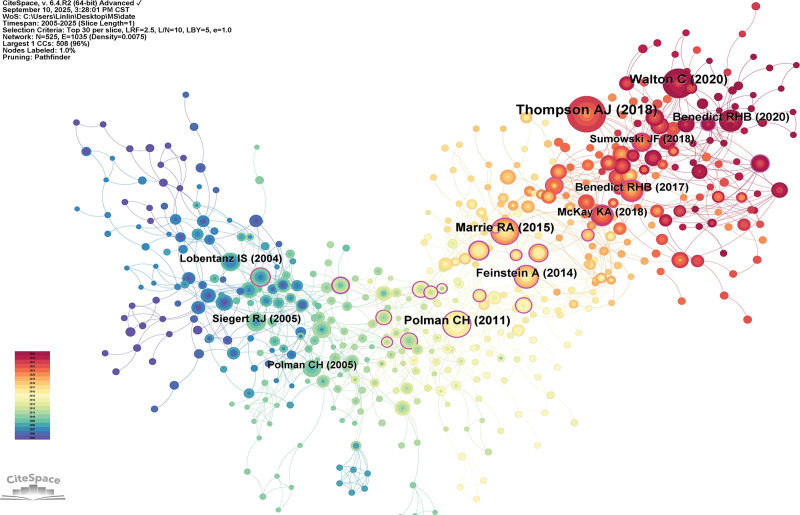
Highly-cited publications and co-cited references. Co-citation network map. Nodes of the same color indicate the same cluster. The deeper the red, the more recent the publication date.

When 2 or more papers are cited by another paper, a connection is established between the cited and co-cited relationships, known as co-citation (Fig. 9). The top 10 most cited documents are shown in Table 3. Three of them are from the MS Journal. This journal holds the highest impact in the field of MS. The most central document is 1 published by Boeschoten RE in the Journal of the Neurological Sciences. It discusses the prevalence of depression and anxiety in MS. Additionally, among the top 10 documents, 2 focus on the prevalence of depression and anxiety in MS, indicating that this is a research hotspot in the field.

**Table 3:** The top 10 highly cited studies.

Rank	Cited reference	Title	Co-citation	Centrality	Year
1	Boeschoten RE, 2017, J Neurol Sci, V372, P331, DOI 10.1016/j.jns.2016.11.067	Prevalence of depression and anxiety in multiple sclerosis: a systematic review and meta-analysis	138	0.22	2017
2	Marrie RA, 2015, Mult Scler J, V21, P305, DOI 10.1177/1352458514564487	The incidence and prevalence of psychiatric disorders in multiple sclerosis: a systematic review	79	0.05	2015
3	Feinstein A, 2014, Nat Rev Neurol, V10, P507, DOI 10.1038/nrneurol.2014.139	The link between multiple sclerosis and depression	59	0.07	2014
4	Benedict RHB, 2017, Mult Scler J, V23, P721, DOI 10.1177/1352458517690821	Validity of the symbol digit modalities test as a cognition performance outcome measure for multiple sclerosis	56	0.13	2017
5	McKay KA, 2018, Neurology, V90, PE1316, DOI 10.1212/WNL.0000000000005302	Psychiatric comorbidity is associated with disability progression in multiple sclerosis	48	0.1	2018
6	Lobentanz IS, 2004, Acta Neurol Scand, V110, P6, DOI 10.1111/j.1600-0404.2004.00257.x	Factors influencing quality of life in multiple sclerosis patients: disability, depressive mood, fatigue and sleep quality	44	0.11	2004
7	Siegert RJ, 2005, J Neurol Neurosur Ps, V76, P469, DOI 10.1136/jnnp.2004.054635	Depression in multiple sclerosis: a review	41	0.07	2005
8	Dobson R, 2019, Eur J Neurol, V26, P27, DOI 10.1111/ene.13819	Multiple sclerosis – a review	41	0.03	2019
9	Benedict RHB, 2005, J Neurol Sci, V231, P29, DOI 10.1016/j.jns.2004.12.009	Predicting quality of life in multiple sclerosis: accounting for physical disability, fatigue, cognition, mood disorder, personality, and behavior change	41	0.16	2005
10	Patten SB, 2003, Neurology, V61, P1524, DOI 10.1212/01.WNL.0000095964.34294.B4	Major depression in multiple sclerosis: a population-based perspective	34	0.06	2003

## 4. Discussion

### 4.1. Current research status

In recent years, the number of publications in the field of MS depression and anxiety has been on a continuous rise, with an accelerated growth since 2013, peaking in 2022. The United States and its institutions, particularly the University of Washington, have published the most papers in this field. The United States accounts for one-third of the total publications, and among the top 10 institutions, 4 are from the United States. On the one hand, the United States’ commanding position stems from massive federal and foundation investment, together with well-established multi-center consortia such as NARCOMS and MS-CQI that accelerate data sharing and publication output. On the other hand, the country’s high MS prevalence (≈288/1,00,000) continuously generates large, clinically well-characterized cohorts, providing a steady stream of research questions and patients for studies. In contrast, many other nations and organizations lack comparable funding, trained personnel, and dedicated infrastructure, making it difficult to produce high-impact findings. At the institutional level, the University of Washington leads the publication count, and European and North American institutes consistently outperform those in other regions in output, *H*-index, and average citations per paper – differences that again mirror disparities in financial resources and length of research tradition. Individually, Marrie, Ruth Ann not only tops the author productivity list but also ranks near the peak in total link strength, *H*-index, and average citations per paper, underscoring her status as one of the most influential researchers in the field.

### 4.2. Research hotspots and trends

Keyword co-occurrence, bursts, and document co-citation suggest changes in research hotspots over time. In recent years, prevalence, mechanisms, cognitive impairment, and exercise have emerged as the current research hotspots in the field of MS depression and anxiety. This alignment coincides with recent breakthroughs in the field: in 2023 the WHO classified depression as a core disability metric for MS, paralleling the keyword burst “epidemiology” (2018–2024, strength 19.7), while exercise interventions were incorporated into Canada’s MS clinical guidelines for the first time, mirroring the “exercise” burst (2020–2024, strength 15.4).

#### 4.2.1. Prevalence

The prevalence of MS depression and anxiety varies significantly across different countries. In the Middle East, for example, the overall prevalence of depression among Iranian^[9]^ MS patients is 47% (95% CI: 39–55%), and the prevalence of anxiety is 51% (95% CI: 36–66%). In Saudi Arabia,^[10]^ 83.1% of female MS patients and 62.1% of male MS patients suffer from depression, and 66.8% of MS patients have anxiety disorders. In Canada,^[11]^ the 2-week incidence of depression among MS patients is 0.019 (95% CI: 0.013–0.029) for females and 0.044 (0.026–0.074) for males.

The significant differences in prevalence rates across countries may be attributed not only to ethnic and cultural differences but also to the heterogeneity of sample sizes, clinical measurements, and assessment methods. The primary tools for assessing MS depression include scales and semi-structured interviews, such as the beck depression inventory and the Hospital Anxiety and Depression Scale. For instance, the study in Saudi Arabia employed the Patient Health Questionnaire 9 and the generalized anxiety disorder 7 questionnaire, while the Canadian study used the Composite International Diagnostic Interview and the Structured Clinical Interview for DSM-IV-TR (SCID). Additionally, some symptoms of MS itself, such as fatigue, sleep problems, and pain, overlap with depressive symptoms, potentially leading to an overestimation of depressive symptoms.^[11]^ On the other hand, there is a notable lack of studies from East Asia, Europe, and Africa in recent years. Therefore, in the field of MS depression and anxiety epidemiology, the use of unified assessment tools and more comprehensive statistics is necessary.

#### 4.2.2. Mechanisms

The etiology of depression and anxiety in MS remains unclear, but research suggests it likely involves multiple factors. Regarding genetic factors it remains controversial whether MS-related depression and anxiety are associated with genes. For example, one study investigated the potential role of the Val66Met polymorphism of brain-derived neurotrophic factor in MS-related depression but found no association between this factor and depression in MS.^[12]^ However, in terms of family history of depression, some studies have detected a higher prevalence of depression in female MS patients with a family history of depression.^[13]^ Immune-inflammatory factors are considered key contributors. Research has shown that MS patients with depression have a higher proportion of T4 helper/inducer cells in their peripheral blood,^[14]^ and this is associated with a higher CD4/CD8 ratio.^[15]^ During relapses, there is an increase in the levels of tumor necrosis factor and interferon messenger RNA in whole blood.^[16]^ These cytokines may trigger depressive symptoms by influencing serotonin synthesis and reuptake, as well as by activating the hypothalamic-pituitary-adrenal axis, leading to increased secretion of adrenocorticotropic hormones.^[17,18]^ Additionally, psychosocial factors, such as the ability to cope with stress, insufficient social support, and the psychological burden of the disease, can all influence the occurrence and development of depression.^[19]^

Furthermore, imaging studies have found that MS-related depression may be associated with multifaceted damage to brain structure and function. MS patients with depression exhibit reduced gray matter volume, particularly in the frontal and temporal lobes, as well as hippocampal atrophy.^[19-22]^ These areas of damage may impair emotional regulation and cognitive function, thereby increasing the risk of depression. Moreover, white matter lesions, such as increased lesion load in T2 and T1 weighted imaging, and reduced white matter integrity in diffusion tensor imaging, indicate impaired neural signal transmission, which may further affect emotional and cognitive function.^[23]^ These factors interact to collectively influence the occurrence and development of depression in MS patients.

#### 4.2.3. Cognitive impairment

Cognitive impairment is a common symptom in MS, affecting approximately 40% to 60% of MS patients.^[24]^ The most frequently affected cognitive domains include processing speed, learning and memory, followed by declines in visual-spatial processing and executive function.^[25]^ In recent years, numerous studies have explored the relationship between depressive symptoms and cognitive performance in MS patients, but the results have been inconsistent. Some studies have observed that MS patients with depressive and anxiety symptoms exhibit poorer cognitive performance.^[26,27]^ However, other studies have not found such an association.^[28,29]^ A meta-analysis conducted by Altieri et al^[30]^ showed that higher levels of depressive symptoms are associated with poorer performance not only in global cognitive tasks but also in several specific cognitive domains. Specifically, depressive symptoms have the strongest correlation with verbal memory, spatial memory, verbal fluency, and inhibitory control, followed by global cognition, attention, processing speed, and working memory, while attentional shifting ability is not significantly related to depressive symptoms.

#### 4.2.4. Exercise

In recent years, exercise as a non-pharmacological intervention has garnered attention. A meta-analysis indicated that both aerobic and resistance exercise training can alleviate depressive symptoms in MS patients.^[31]^ However, the current research findings are not consistent, with only some studies showing that exercise can significantly improve depressive symptoms, while others do not observe a significant effect.^[32,33]^ This discrepancy may be related to various factors, including the baseline level of depression, disability level, intensity and type of exercise, and the sensitivity of the depression assessment tools used.^[34]^ For example, a meta-analysis by Adamson^[35]^ found that interventions that meet physical activity guidelines have twice the effect on reducing depression compared to those that do not meet the guidelines. Therefore, there is still a significant amount of work needed to explore exercise prescriptions based on depression levels in MS.

## 5. Conclusion

The results of the bibliometric analysis provide valuable insights into the current research landscape and developmental trends. Research related to depression and anxiety in MS is gradually increasing. Collaboration among different countries, institutions, and authors is also on the rise. However, collaboration between countries and institutions has regional characteristics, with less collaboration between Asia and Europe and America. Based on the analysis, we predict that the prevalence, etiology, relationship with cognitive impairment, and exercise therapy of MS depression and anxiety may be the hotspots in this field.

## Author contributions

**Conceptualization:** Rongrong Yuan.

**Data curation:** Linlin Zhang, Qin Yang.

**Formal analysis:** Rongrong Yuan, Qin Yang.

**Investigation:** Qin Yang.

**Software:** Linlin Zhang, Qin Yang.

**Validation:** Rongrong Yuan.

**Visualization:** Rongrong Yuan.

**Writing – original draft:** Rongrong Yuan.

**Writing – review & editing:** Linlin Zhang, Qin Yang.
